# An Inducible TGF-β2-TGFβR Pathway Modulates the Sensitivity of HNSCC Cells to Tyrosine Kinase Inhibitors Targeting Dominant Receptor Tyrosine Kinases

**DOI:** 10.1371/journal.pone.0123600

**Published:** 2015-05-06

**Authors:** Emily K. Kleczko, Jihye Kim, Stephen B. Keysar, Lydia R. Heasley, Justin R. Eagles, Matthew Simon, Marianne E. Marshall, Katherine R. Singleton, Antonio Jimeno, Aik-Choon Tan, Lynn E. Heasley

**Affiliations:** 1 Department of Craniofacial Biology, University of Colorado Anschutz Medical Campus, Aurora, Colorado, United States of America; 2 Department of Medicine, University of Colorado Anschutz Medical Campus, Aurora, Colorado, United States of America; 3 Veterans Affairs Eastern Colorado Healthcare System, Denver, Colorado, United States of America; Winship Cancer Institute of Emory University, UNITED STATES

## Abstract

The epidermal growth factor receptor (EGFR) is overexpressed in approximately 90% of head and neck squamous cell carcinomas (HNSCC), and molecularly targeted therapy against the EGFR with the monoclonal antibody cetuximab modestly increases overall survival in head and neck cancer patients. We hypothesize that co-signaling through additional pathways limits the efficacy of cetuximab and EGFR-specific tyrosine kinase inhibitors (TKIs) in the clinical treatment of HNSCC. Analysis of gene expression changes in HNSCC cell lines treated 4 days with TKIs targeting EGFR and/or fibroblast growth factor receptors (FGFRs) identified transforming growth factor beta 2 (TGF-β2) induction in the three cell lines tested. Measurement of TGF-β2 mRNA validated this observation and extended it to additional cell lines. Moreover, TGF-β2 mRNA was increased in primary patient HNSCC xenografts treated for 4 weeks with cetuximab, demonstrating in vivo relevance of these findings. Functional genomics analyses with shRNA libraries identified TGF-β2 and TGF-β receptors (TGFβRs) as synthetic lethal genes in the context of TKI treatment. Further, direct RNAi-mediated silencing of TGF-β2 inhibited cell growth, both alone and in combination with TKIs. Also, a pharmacological TGFβRI inhibitor similarly inhibited basal growth and enhanced TKI efficacy. In summary, the studies support a TGF-β2-TGFβR pathway as a TKI-inducible growth pathway in HNSCC that limits efficacy of EGFR-specific inhibitors.

## Introduction

Worldwide, head and neck squamous cell carcinoma (HNSCC) is the 6^th^ most common cancer [1,2]. While the morbidity of the disease has decreased due to better organ preservation surgeries [3], the overall five-year survival rate for HNSCC has not improved significantly in the past several decades, remaining at 40–50% [4,5]. Thus, it is imperative to develop new therapies to improve survival. The modern approach to personalized cancer therapeutics involves identifying the dominant growth pathway(s) in cancer cells and subsequently treating with an inhibitor of this pathway. In this regard, the epidermal growth factor receptor (EGFR) is overexpressed, but rarely mutated [6,7], in about 90% of HNSCC tumors [4,8], making it an attractive target for therapy. Both monoclonal antibodies, such as cetuximab, and tyrosine kinase inhibitors (TKIs), such as gefitinib and erlotinib, have been clinically tested in HNSCC [9–11]. EGFR-targeted therapy alone has not yielded cures [11,12], but when combined with radiotherapy, cetuximab improved the median survival from 29.3 months to 49 months [13].

Many factors may account for the limited effects of EGFR-targeted therapy, including intrinsic and acquired resistance to these drugs. Recently, our group demonstrated that the fibroblast growth factor receptor (FGFR) pathway functions as a dominant driver in a subset of HNSCC cell lines that are inherently insensitive to EGFR-specific TKIs [14]. Thus, EGFR inhibitor insensitivity is, in part, mediated by the functioning of alternative driver pathways. Additionally, acquired resistance has become an increasingly apparent problem in treating various cancers with targeted therapies. For example, in non-small cell lung cancer (NSCLC), resistance to EGFR-selective TKIs occurs via gatekeeper mutations in EGFR, selection for MET amplification, and perhaps other mechanisms including the induction of FGFR-dependent bypass pathways [15–18]. In HNSCC, neither primary driver mutations nor gatekeeper mutations are observed at significant frequencies in EGFR [19,20]. However, other mechanisms of resistance have been reported in HNSCC, including increased expression of cyclin D1 [21,22].

In this study we deployed complementary approaches to identify signaling pathways that reduce the efficacy of EGFR targeting inhibitors in HNSCC. Gene expression analysis of HNSCC cell lines treated for 4 days with EGFR or FGFR-specific TKIs in an FGFR1-dependent cell line revealed TGF-β2 induction. Moreover, a functional genomics approach identified TGF-β2 and TGF-β receptors (TGFβRs) as putative synthetic lethal targets in the setting of TKI treatment in HNSCC cells. Pharmacological and molecular techniques validated TGF-β2 signaling as a growth pathway that regulates the sensitivity to EGFR and FGFR inhibitors in HNSCC cell lines.

## Materials and Methods

### Cell Culture

HNSCC cells used in these studies were previously described [14,23] and grown in Dulbecco’s modified Eagle’s medium (DMEM) (UMSCC25, UMSCC8, HN31) or Roswell Park Memorial Institute-1640 (RPMI-1640) (Ca9-22 and 584-A2) growth media (Invitrogen, Carlsbad, CA) supplemented with 10% fetal bovine serum and 1% penicillin-streptomycin (Sigma-Aldrich, St. Louis, MO); cells were grown in a humidified incubator with 5% CO_2_ at 37°C. All HNSCC cell lines used for investigations herein were submitted for DNA fingerprinting to verify authenticity.

### Affymetrix Microarray Analysis

UMSCC25, 584-A2, and Ca9-22 HNSCC cells were treated with dimethylsulfoxide (DMSO) as a control, 0.3μM FGFR-TKI, AZD8010, 0.1μM EGFR-TKI, gefitinib, or both AZD8010 and gefitinib for 4 days after which RNA was purified and used to probe Affymetrix Human Gene 1.0 ST arrays by the Genomics and Microarray Core, University of Colorado Cancer Center Gene Expression Core. Gene expression profiles were extracted and normalized by using Robust Multiarray Average (RMA) using Affymetrix Power Tools. Data analysis was performed on these normalized gene expression profiles.

### RNA Interference-Based Functional Genomics Screens

#### Lentivirus Preparation

The MISSION TRC1 human kinome shRNA library (Functional Genomics Shared Resource, University of Colorado Cancer Center) was packaged in 293T cells with packaging component vectors coding for p-CMV-VSV-G and p8.9. Cells were incubated overnight with Turbofect transfection reagent (Fermentas, Glen Burnie, MD), packaging vectors, and the shRNA library. The lentiviruses released into the medium were filtered using a 0.45-μm filter (Corning Inc., Corning, NY). 6x10^6^ cells from each of the cell lines (UMSCC25, UMSCC8, UMSCC1) were plated into duplicate 15cm plates (3x10^6^ cells/plate) and transduced 24 hours later with the kinome shRNA library containing viral supernatant and 8 μg/ml polybrene (Sigma-Aldrich). After 72 hours, transfected cells were selected with puromycin (1 μg/ml) for 5 days. The lentiviral preparation for the SBI genome-wide screen was reported in our previous publication [24].

#### Cell Line Treatment and shRNA Sequencing

Library expressing HNSCC cells were divided into nine groups of 7.5x10^5^ cells per 15cm plate. Three plates were treated with DMSO vehicle, three with 30nM AZD8931 (a pan-ErbB inhibitor), and three with 300nM of the EGFR-specific TKI gefitinib for 72 hours. Subsequently, the media was replaced with drug-free media for an additional 72 hours. Cells were harvested and total genomic DNA (gDNA) was isolated using the Quick-gDNA MiniPrep kit (Zymo Research Corporation, Irvine, CA). The isolated gDNA was submitted to PCR using Takara PrimeSTAR HS DNA polymerase (Clontech Laboratories, Mountain View, CA) and forward primer, 5’-GGA CTA TCA TAT GCT TAC CGT AAC-3’ and the reverse primer, 5’-CCA AAG TGG ATC TCT GCT GTC CC-3’. The PCR product was purified using the QIAquick PCR Purification Kit (Qiagen), and submitted to digestion with the *Xho*I (Fermentas) restriction enzyme. Following desalting of the DNA using QIAEX II Gel Extraction Kit (Qiagen), barcode linkers were ligated to the samples using T4 DNA ligase (Fermentas) and then submitted to a second round of PCR to add the Illumina adapter sequences using Phusion high fidelity DNA polymerase (New England Biolabs, Ipswich, MA), and primer sequences 5’-CAAGCAGAAGACGGCATACGATGGAAAGGACGAAACACCGG-3’ and 5’-AATGATACGGCGACCACCGAGATCTACACTCTTTCCCTACACGACGCTCTTC CGATCT-3’. The samples were purified using QIAquick PCR Purification Kit (Qiagen) and sequenced with an Illumina Genome Analyzer IIx (Illumina, San Diego, CA).

#### Bioinformatic Analysis

The BiNG!S bioinformatics method for analyzing the functional genomics shRNA sequencing reads has previously been published [24,25]. The data set from the SBI genome-wide screen was previously published and deposited in the National Center for Biotechnology Information Gene Expression Omnibus database (accession no. GSE39305) [24].

### Clonogenic Growth Assays

Cells were seeded in 6-well plates at 100 cells/well in full media. After 24 hours, growth media was changed and drugs were added. Fresh media with drugs was added weekly. After 2–3 weeks, the plates were rinsed with phosphate buffered saline (PBS), and the colonies were fixed and stained with a 6% (vol/vol) gluteraldehyde and 0.5% (wt/vol) crystal violet solution for 30 minutes at room temperature. Following rinsing with water, the wells were photographed and the images were quantified with the MetaMorph software program (Molecular Devices, Downington, PA) to quantify the total area of the colonies in each well. Data are represented as either “Total Colony Area” or “Total Colony Area (Fold Change)” if the data were normalized to the DMSO control.

### TGF-β2 ELISA

Cells were seeded in 12-well plates at 50,000 cells/well. 24-hours later, the cells were treated for 4 days with DMSO vehicle control, AZD8010 (300nM), gefitinib (100nM), or the combination of TKIs. The media was collected and assayed for TGF-β2 according to the manufacturer’s instructions (Quantikine human TGF-β2 ELISA kit; R&D Systems, Minneapolis, MN). The concentration of TGF-β2 in the media was normalized to the total cellular protein per well, and the data are presented as pg of TGF-β2/mg cell protein.

### Quantitative Real-Time Polymerase Chain Reaction (qRT-PCR)

Total RNA was isolated from HNSCC cells using the RNeasy Mini Kit (Qiagen, Germantown, MD). Aliquots containing 5μg of RNA were reversed transcribed to cDNA following the Fermentas Maxima Kit Protocol (Fermentas, Glen Burnie, MD), after which the cDNA was diluted 1:25 in water. Aliquots (5μl) were submitted to quantitative PCR with SYBR green Jumpstart Taq Readymix (Sigma-Aldrich) plus primers for either TGF-β1 or TGF-β2 using a MyiQ real time-PCR detection system (BioRad, Hercules, CA). Forward primer 5’-TTG AGG GCT TTC GCC TTA GC-3’ and reverse primer 5’-AAC CCG TTG ATG TCC ACT TGC-3’ were used to detect TGF-β1 levels. The TGF-β2 primers used were forward 5’-ATC TGC TTC TCC CTG CGT-3’ and reverse 5’-GCT GTT CAA TCT TGG GTG TTT TGC-3’. Forward primer 5’-TGA TAA AAC TTG CTC TGT CCA CGG-3’ and reverse primer 5’-AAT GGC TGG CTT TCC TTG GG-3’ were used to assess mRNA levels of TGFβRI. Forward primer 5’-TGG AGT TCA GCG AGC ACT GTG-3’ and reverse primer 5’-TGT TGT GGT TGA TGT TGT TGG C-3’ were used to assess mRNA levels of TGFβRII. Levels of mRNAs were normalized to the GAPDH mRNA levels that were measured in replicate samples, and data are presented as “Relative Expression Levels”.

### Luciferase Reporter Assays

In 6-well plates, HNSCC cells were transfected with 2.5μg of a promoter-driven firefly luciferase plasmid and 2.5μg TK-Renilla using TransIT-2020 transfection reagent (Mirus, Madison, WI) according to the manufacturer’s protocol. Cells were treated with inhibitors (0.3μM AZD8010, 0.1μM gefitinib, 1μM SB525334 (Tocris Bioscience, Minneapolis, MN)) for 72 hours after which the cells were lysed and assayed following the protocol for the Dual-Luciferase Reporter Assay System (Promega, Madison, WI). The TGF-β2 promoter luciferase construct was obtained from SwitchGear Genomics (Menlo Park, CA).

### Patient-Derived Xenograft (PDX) HNSCC Tumors

The generation of the HNSCC PDX tumors and their treatment were previously reported [26]. Briefly, tumors were implanted into the flanks of nu/nu mice, and once tumors reached an appropriate volume, the mice were treated with vehicle control or 40mg/kg of cetuximab twice a week by IP for 4 weeks. Total RNA was isolated from PDX tumors by homogenization using the QIAzol Lysis Reagent (Qiagen), and then the samples were further purified using the RNeasy Mini Kit (Qiagen).

### shRNA-Mediated Gene Silencing

Lentiviral-encoded shRNAs from the TRC set containing sequences targeting two different regions of TGF-β2, shTGFB2.4 (TRC0000033427) and shTGFB2.5 (TRC0000033428), and a non-silencing control (NSC) shRNA that targets green fluorescence protein (GFP) were obtained through the Functional Genomics Shared Resource, University of Colorado Cancer Center. The lentiviral vector backbone for these sequences is pLKO.1-puro. 293T cells were used to package the lentiviruses encoding the shRNAs by co-transfection with p-CMV-VSV-G and pΔ8.9. UMSCC25 cells were incubated with the lentiviruses in conditioned media and then submitted to selection with puromycin (1μg/mL, Sigma-Aldrich), and pooled puromycin-resistant cultures were used for the experiments.

### Proliferation Assays

Effects of drug treatment on cell proliferation were assessed with the CyQUANT Direct Cell Proliferation Assay (Invitrogen). Cells were seeded in 96-well plates at 100 cells/well. The next day, fresh medium containing gefitinib and SB525334 at concentrations ranging from 0–100 nM was added. The cells were incubated for 7 days after which the plates were developed and read on a plate reader following the manufacturer’s protocol. DMSO was used as the vehicle control, and the experiments were performed in duplicate. The experiment was also performed in the presence or absence of 300nM AZD4547. The combinatorial index (CI) values were calculated using the CalcuSyn Software (Biosoft, Cambridge, UK).

### Immunoblotting

Cells were treated for 4 days with DMSO-control, 0.1μM gefitinib, 0.3μM AZD4547, or the combination of both TKIs. For protein isolation, cells were rinsed in phosphate buffered saline (PBS) and collected by spinning them at 1,500 x g for 5 minutes. Cells were then lysed in MAP Kinase Lysis Buffer (MKLB) containing 0.5% Triton X-100, 50 mM β-glycerophosphate (pH 7.2), 0.1 mM Na3VO4, 2mM MgCl2, 1 mM EGTA, 1mM DTT, 0.3 M NaCl, 2 μg/mL leupeptin and 4μg/mL aprotinin. The samples were centrifuged at 13,000 x g for 5 minutes and the pellet was discarded while the supernatant was kept. Protein lysates were submitted to SDS-PAGE. After electrophoretic transfer onto a nitrocellulose filter, filters were blocked in 3% bovine serum albumin (BSA) (Cohn Fraction V, ICN Biomedicals, Inc., Aurora, OH) in Tris-buffered saline with 0.1% Tween 20 (TBST) for 1 hour. Filters were then incubated overnight at 4°C with primary antibodies. The following day, filters were treated washed 3 times in TBST and then incubated for 1 hour with alkaline-phosphatase-coupled goat anti-rabbit or mouse antibodies. Following this, filters were washed in TBST and developed with LumiPhos reagent (Pierce, Rockford, IL) according to the manufacturer’s instructions. Filters were stripped and reprobed for with loading controls.

For analysis of phospho-Smad2 and total Smad2/3 expression, aliquots of cell lysates containing 75μg of protein were submitted to SDS-PAGE and immunoblotted for phospho-Smad2 (S465/467) (#3101, Cell Signaling Technology) and reprobed for total Smad2/3 levels (#3102, Cell Signaling Technology). The Na^+^/K^+^-ATPase α subunit (sc-21712) (Santa Cruz Biotechnology, Inc.) was used as a loading control.

### Statistical Analysis

Unless otherwise noted, data are the mean and standard error of the mean (SEM) of three independent experiments performed in triplicate. The Prism program was used to make graphs, and a student’s unpaired t-test was performed on data to determine statistical significance. “ns” denotes not significant, and * indicates a significant p-value, which is defined in each figure legend. For evaluation of CI values, the CalcuSyn Software was utilized.

## Results

### Induction of TGF-β2 in Response to EGFR and FGFR-Specific TKIs

**Fig 1:**
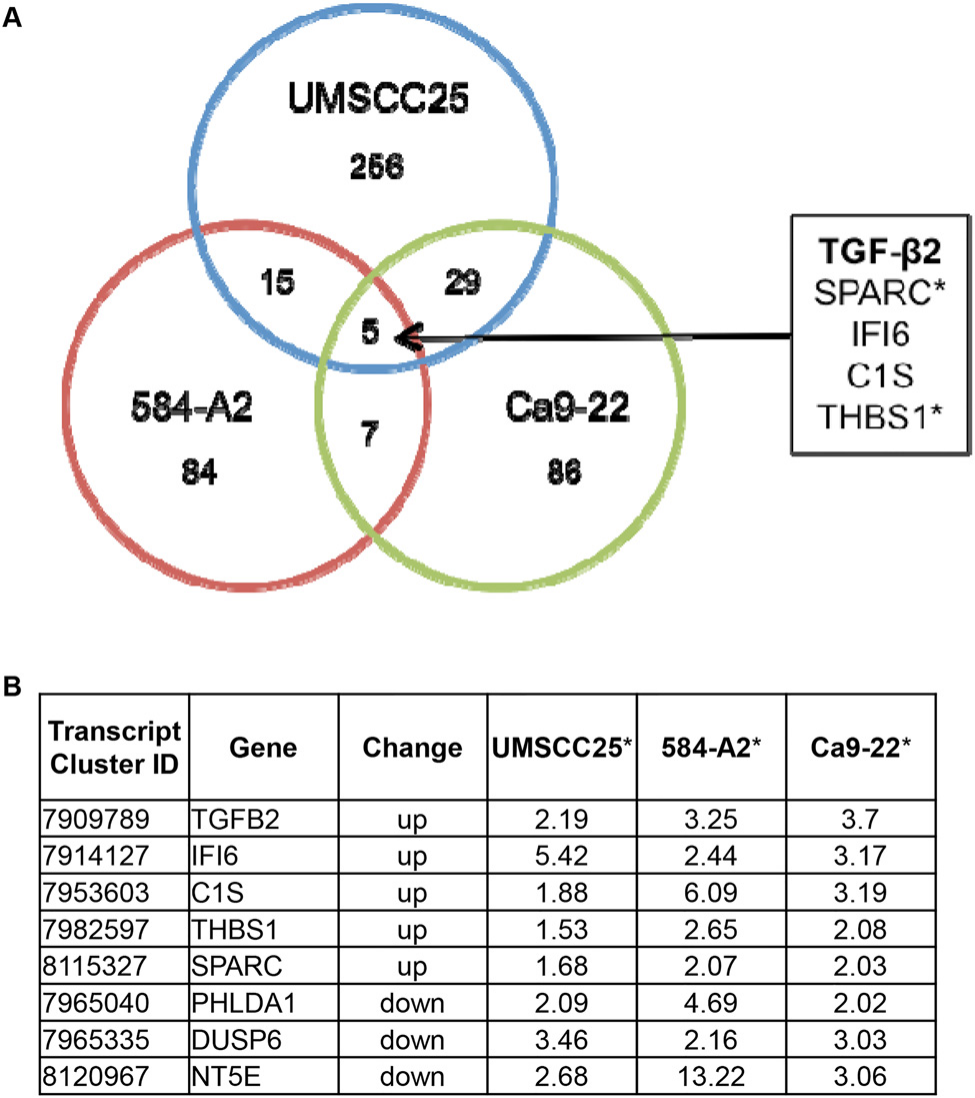
Comparison of genes upregulated after AZD8010 and Gefitinib treatment. RNA and protein was isolated from UMSCC25, 584-A2, and Ca9-22 HNSCC cell lines that were treated for 4 days with DMSO (control), 0.3M AZD8010, 0.1M gefitinib, or both AZD8010 and gefitinib. RNA was submitted to an Affymetrix GeneChip analysis and revealed 5 genes that were upregulated in common with AZD8010 and gefitinib. The genes identified had either a >2 (UMSCC25 and Ca9-22) or >1.5 (584-A2) fold change in expression levels compared to the DMSO control. **A.** A venn diagram showing the genes upregulated in all the cell lines. **B.** Tabulated values for the genes increased and decreased in the AZD8010 and gefitinib treated group. *Numbers are the fold change in the treated compared to the control. TKIs: AZD8010 is an FGF receptor inhibitor and gefitinib is an EGF receptor inhibitor.

Our lab previously categorized a panel of HNSCC cell lines for growth dependence on EGFR or FGFR pathways [14]. Gene expression changes were determined by Affymetrix microarray analysis in HNSCC cells treated for 4 days with AZD8010, an FGFR-specific TKI [18], gefitinib, an EGFR-specific TKI, or the combination of both TKIs. The cell lines used in the experiment were sensitive to both EGFR and FGFR-specific TKIs (UMSCC25 and Ca9-22) or only FGFR-TKIs (584-A2) [14]. Analysis of the resulting data identified 5 genes that were upregulated in all three cell lines after treatment with TKIs, and included TGF-β2 (Fig 1). In addition, thrombospondin-1 (THBS1), a multifunctional protein reported to promote activation of latent TGF-β2 [27,28], and secreted protein acidic and rich in cysteine (SPARC), shown to regulate TGF-β signaling [29] and identified as a THBS1 interactor, were increased in all three cell lines. Based on the data supporting increased TGF-β2 activity, we pursued this pathway in more detail.

**Fig 2:**
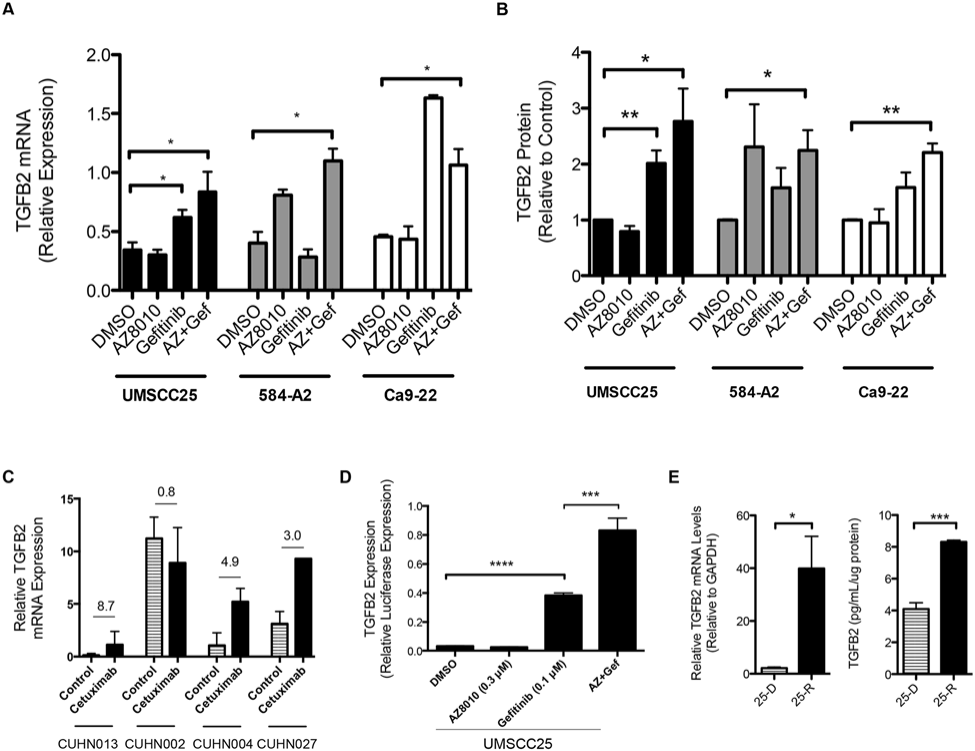
TGF-β2 is induced in response to treatment with EGFR and FGFR TKIs. **A.** qRT-PCR was performed to verify that TGF-2 mRNA was upregulated after TKI treatment in the cell lines used for the gene chip (UMSCC25, 584-A2, Ca9-22). *denotes a p-value <0.05. **B.** An ELISA was performed on the media from the cell lines and revealed an increase in TGF-2 protein secreted from cells upon treatment with TKIs. **C.** Patient-derived xenograft tumors were treated for 4 weeks with vehicle control or cetuximab, after which RNA was isolated from the tumors and qRT-PCR was performed to assess TGF-β2 mRNA levels. The values indicate the fold change between the treated and control tumors. **D.** A TGF-β2 promoter firefly luciferase construct was used to measure changes in TGF-β2 transcription in UMSCC25 cells in response to EGFR and FGFR TKIs. This experiment is a representative of 3 others. In all experiments, cells were treated for 4 days with DMSO, 0.1μM gefitinib, 0.3μM AZD8010, or AZD8010 and gefitinib. **E.** UMSCC25 cells were adapted to increasing concentrations of gefitinib (up to 3μM) over the course of 3 months. RNA was isolated and qRT-PCR was performed to determine the mRNA levels of TGF-β2. An increase in TGF-β2 mRNA is observed in the chronically adapted cells (25-R) versus the control cells (25-D). Data are the average of the percent of control of 3 independent RNA samples. An ELISA shows that the concentration of TGF-β2 in the media of 25-R cells is greater than the concentration in the media from control cells. *denotes a p-value <0.025; **denotes a p-value <0.007; ***denotes a p-value <0.0005.

Quantitative real-time PCR (qRT-PCR) and an ELISA confirmed TGF-β2 mRNA and protein induction following treatment with TKIs that inhibit the dominant growth pathways in each cell line (Fig 2A and 2B). Specifically, gefitinib is dominant in the dual cell lines, UMSCC25 and Ca9-22, but the FGFR-specific TKI increases this response further, though not statistically significant from gefitinib alone. By contrast, the FGFR-dominant cell line, 584-A2, shows a TGF-β2 induction only with AZD8010. The microarray data revealed that neither TGF-β1 nor TGF-β3 were induced in response to TKI treatment, and this was confirmed for TGF-β1 by qRT-PCR. This is consistent with the differing regulation of the TGF-β1, TGF-β2, and TGF-β3 promoters [30]. Thus, induction of TGF-β2 expression is a general finding in HNSCC cell lines following treatment with inhibitors of EGFR and FGFRs. Further, expression of TGFβRI and TGFβRII by quantitative RT-PCR was detectable in all the cell lines used in this study (S1 Fig).

To test for TGF-β2 mRNA induction in a distinct set of HNSCC patient-derived xenografts (PDX), we obtained RNA from 4 HNSCC PDX models (CUHN002, CUHN004, CUHN013, CUHN027) that were propagated in nu/nu mice and treated for 4 weeks with vehicle control or the EGFR monoclonal antibody, cetuximab. All PDX tumors responded to cetuximab treatment with a reduction or stabilization of tumor size [26]. TGF-β2 mRNA was increased in 3 of the 4 PDXs (CUHN013, CUHN004, and CUHN027) with fold changes of 8.7, 4.9, and 3.0, respectively (Fig 2C). These data indicate that induction of TGF-β2 in response to inhibiting EGFR is not limited to *in vitro* experiments with cell lines, but also observed with PDX models treated *in vivo*. Thus, TGF-β2 induction could be an important alternative driver pathway in HNSCC tumors.

To determine if TGF-β2 induction was related to transcriptional activation, a reporter plasmid containing the TGF-β2 promoter linked to firefly luciferase (TGFB2-luc) was transfected into UMSCC25 cells followed by treatment with DMSO, 0.3μM AZD8010, 0.1μM gefitinib, or the combination of the drugs for 3 days. As shown in Fig 2D, treatment with EGFR and FGFR TKIs increased TGF-β2-luciferase activity, indicating that elevated TGF-β2 mRNA and protein levels following TKI treatment resulted from increased gene transcription.

We recently showed rapid induction of FGFR2 and FGFR3 expression in lung cancer cell lines following treatment with EGFR-specific TKIs [15,18]. To determine if the rapid induction of TGF-β2 is maintained with prolonged TKI exposure, UMSCC25 cells were chronically adapted to increasing gefitinib concentrations until the cells could be continuously cultured in 3μM gefitinib; control cells were simultaneously treated with DMSO [16,31]. Both TGF-β2 mRNA and protein were significantly increased in UMSCC25 cells rendered resistant to gefitinib (Fig 2E). Again, no change in TGF-β1 expression was detected (data not shown). Thus, the rapid increase in TGF-β2 expression that is observed following 4 days of TKI treatment persists after chronic adaptation to TKIs, indicating that the TGF-β pathway may play an important role in the response of HNSCC cells to inhibitors of dominant receptor tyrosine kinase (RTK) pathways.

### Functional Genomics Identifies the TGFβR Pathway as Synthetic Lethal (SL) with TKIs

As a complementary approach to screen for pathways that interact with EGFR and FGFR to drive growth and thereby effect intrinsic sensitivity to TKIs, HNSCC cells were transduced with a genome-wide shRNA library (UMSCC25) or a kinome-targeting shRNA library (UMSCC25 and UMSCC8) and selected for resistance to puromycin as described in the Materials and Methods. Subsequently, the library-expressing cells were treated with or without the TKIs shown in Table 1 and abundance of the transduced shRNAs was defined by massively-parallel deep sequencing. Specific shRNA sequences that are reduced in abundance in cells treated with TKIs are likely to silence genes that provide growth and survival signaling when EGFR or FGFRs are inhibited. By definition, these genes are referred to as synthetic lethal (SL) with respect to these TKIs. As shown in Table 1, TGF-β2 was identified as an SL hit in UMSCC25 cells using the genome-wide shRNA library. In addition, TGFβRI was identified as a high-ranking SL hit in UMSCC25 cells with both gefitinib and the pan-ErbB inhibitor, AZD8931 [32], and TGFβRII was a identified in both UMSCC25 and UMSCC8 cells. Combined, the findings support a hypothesis that an inducible TGF-β2-TGFβR pathway functions to control the sensitivity of HNSCC cells to TKIs targeting dominant RTK pathways.

**Table 1:** Functional genomic screens reveal TGF- pathway is synthetic lethal with TKIs.

Cell Line	shRNA library	TKI	shRNA	GeneSymbol	Control 1	Control 2	Control 3	Treated 1	Treated 2	Treated 3	p value	weightZ_P	Rank	E value
					shRNA count									
UMSCC25	SBI genome wide	AZD8010	SBIGeneNet115533	TGFB2	614	752	2421	225	38	232	0.0075	0.0103	701	7.210
			SBIGeneNet115536	TGFB2	82	30	24	15	11	16	0.118			
UMSCC25	TRC1 kinome library	gefitinib	TRCN0000039774	TGFBRI	3	0	17	0	0	0	9.47E-05	0.00016	20	0.003
			TRCN0000039776	TGFBRI	50	43	23	30	40	7	0.45			
			TRCN0000040011	TGFBRII	52	54	73	26	44	16	0.088	0.21811	219	47.800
			TRCN0000040008	TGFBRII	8	6	4	0	10	1	0.583			
UMSCC25	TRC1 kinome library	AZD8931	TRCN0000039777	TGFBRI	100	48	107	12	19	58	0.0062	0.0047	32	0.149
			TRCN0000039775	TGFBRI	50	28	39	15	16	23	0.049			
			TRCN0000040011	TGFBRII	52	54	73	34	21	20	0.011	0.023	112	2.590
			TRCN0000040008	TGFBRII	8	6	4	6	5	0	0.298			
UMSCC8	TRC1 kinome library	gefitinib	TRCN0000040011	TGFBRII	47	48	97	19	21	2	0.0016	8.84E-05	12	0.001
			TRCN0000040008	TGFBRII	150	36	32	11	37	17	0.027			

### shRNA Silencing TGF-β2 Enhances Growth Inhibition by EGFR and FGFR-Specific TKIs

**Fig 3:**
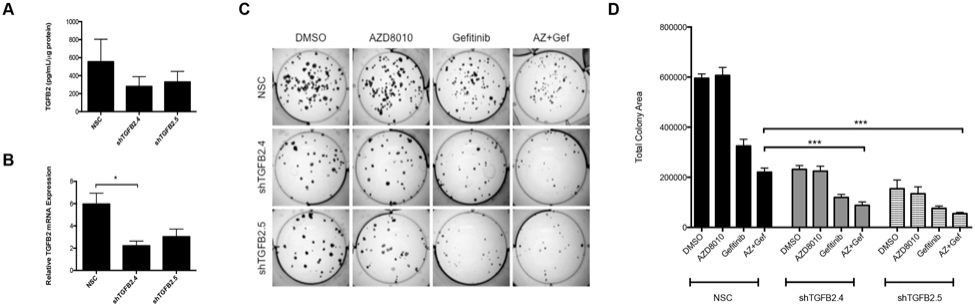
RNAi-mediated silencing of TGF-2 inhibits clonogenic growth in UMSCC25 cells. 2 independent shRNA sequences targeting TGF-β2 or a non-silencing control were transduced into UMSCC25 cells using a lentiviral construct to create 3 cell lines: shTGFB2.4, shTGFB2.5, and NSC. **A.** qRT-PCR was performed to demonstrate that TGF-β2 mRNA was decreased with the shRNA compared to the NSC. **B.** An ELISA performed on media from the knockdown cells demonstrates decreased TGF-β2 protein secretion into the media. **C.** Representative images of the clonogenic assay where cells were treated with DMSO, 0.3μM AZD8010, 0.1μM gefinitib, or both for 2 weeks. **D.** Graphical presentation of the quantified results from the clonogenic assay. ***denotes a p-value <0.0001.

To test the hypothesis that TGF-β2-TGFβR signaling collaborates with specific RTKs to drive growth of HNSCC cells, we used lentiviral-transduced shRNAs to stably silence TGF-β2 in UMSCC25 cells. UMSCC25 cells were infected with lentiviral constructs encoding 2 independent shRNAs targeting TGF-β2 (shTGFB2.4 and shTGFB2.5) or a non-silencing control shRNA targeting GFP (NSC). In pooled cultures of puromycin-resistant cells, a 50–70% reduction in TGF-β2 mRNA (Fig 3A) and protein (Fig 3B) levels were observed. To measure the effect of TGF-β2 silencing on the growth of UMSCC25 cells, a clonogenic assay was performed where the NSC and knockdown cells were treated with DMSO, 0.1μM gefitinib (EGFR-TKI), 0.3μM AZD8010 (FGFR-TKI), or the combination of the two TKIs. Reduction in TGF-β2 levels with the shRNAs led to a 70% decrease in control colony growth compared to the NSC treated with DMSO (Fig 3D). S2 Fig shows the effects of growth inhibition with gefitinib and the shRNAs; the shRNAs further decrease clonogenic growth compared to gefitinib alone. Additionally, the combination of EGFR and FGFR TKIs in the setting of TGF-β2 silencing further decreased growth (p<0.0001) compared to the TKI-treated NSC cells (Fig 3D). These results indicate that TGF-β2 signaling is important for basal growth rates of UMSCC25 cells and combined inhibition of TGF-β2 signaling with EGFR and FGFR TKIs yields greater growth inhibition.

### A TGF-β Receptor Inhibitor Enhances Growth Inhibition by EGFR and FGFR TKIs

**Fig 4:**
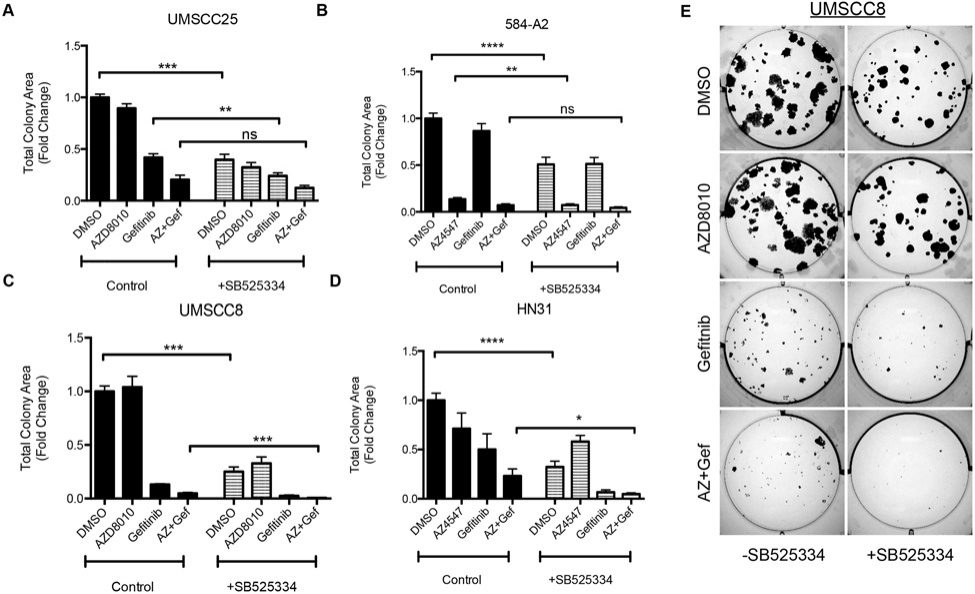
TGF- receptor inhibitors augment the effects of TKIs on colony growth formation. Clonogenic assays were performed where cells were treated with DMSO, 0.3μM AZD8010/AZD4547, 0.1μM gefitinib, or both in the presence or absence of a TGF- receptor I (TGFRI) small molecule inhibitor: SB525334 (1μM). After 2 weeks of incubation, colony growth was measured. This assay was performed in a panel of cell lines and is shown here for **A.** UMSCC25 **B.** 584-A2 **C.** UMSCC8 and **D.** HN31. **E.** Representative images of the experiment performed with UMSCC8 cells in C. *denotes a p-value <0.05; **denotes a p-value <0.006; *** and ****denotes a p-value <0.0001.

**Fig 5:**
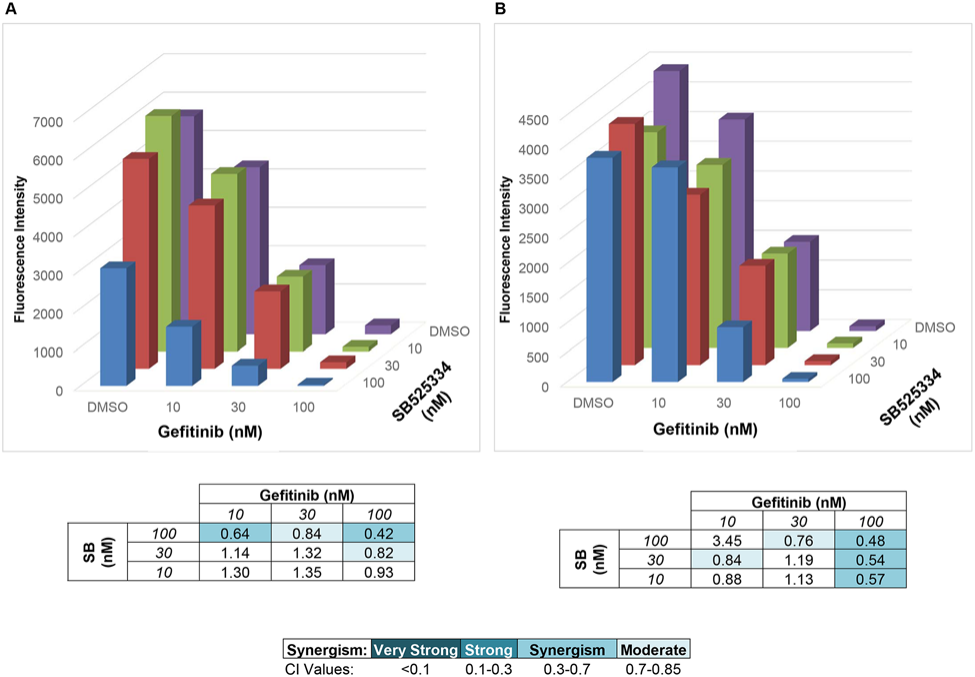
TKIs and SB525334 synergistically inhibit proliferation of UMSCC25 cells. A CyQUANT Direct Cell Proliferation Assay was performed on UMSCC25 cells treated with a concentration range of gefitinib and SB525334, alone and in combination. The experiment was performed in the absence (**A.**) or presence (**B.**) of 0.3μM AZD4547. The combinatorial indices (CI) were calculated with CalcuSyn Software and are presented in the tables below each graph.

The synthetic lethal screens identified TGFβRI and TGFβRII as a significant hit in cells treated with EGFR or FGFR-specific TKIs (Table 1). We used SB525334, a TGFβRI inhibitor [33] to pharmacologically test the role of the TGF-β pathway in 4 HNSCC cell lines. Signaling through the TGF-β pathway requires heterodimerization of TGFβRII and TGFβRI [34]; thus, inhibiting one of the receptors is sufficient to block downstream signaling. Clonogenic assays were performed where cells were treated with DMSO, AZD8010, gefitinib, or the combination of the two TKIs in the presence or absence of SB525334. Consistent with TGF-β2 silencing (Fig 3C), SB525334, alone, significantly reduced clonogenic growth of all four cell lines (Fig 4). Furthermore, the triple combination of geftinib, the FGFR-TKI AZD4547, and SB525334 inhibited growth more than the TKIs alone (Fig 4). While gefitinib exerted no effect on the FGFR-dependent cell line, 584-A2, the combination of AZD4547 and SB525334 yielded greater growth inhibition than AZD4547 alone (Fig 4B). Similarly, the combination of gefitinib, AZD4547, and SB525334 yielded greater inhibition of UMSCC8 and HN31 cell growth than gefitinib and AZD4547 alone (Fig 4C). While addition of SB525334 to gefitinib and AZD4547 did not yield significantly greater clonogenic growth inhibition in UMSCC25 cells (Fig 4A), more extensive analysis of the interaction over multiple drug concentrations demonstrated synergistic inhibition of UMSCC25 cell proliferation with specific combinations of gefitinib and SB525334 (Fig 5A) or gefitinib, AZD4547 and SB525334 (Fig 5B). Thus, the TGF-β-TGFβR pathway plays an important growth role in HNSCC cell lines and collaborates with the EGFR and FGFR pathways for driving transformed growth.

## Discussion

The structure and function of the TGF-β signaling pathway is clearly complex, and has been shown to be both tumor-promoting and tumor-suppressive depending on the context and stage of tumor development [34–36]. During tumor initiation and early progression, the TGF-β pathway is viewed as a tumor suppressor with anti-proliferative effects [37]. During later stages of tumorigenesis, the TGF-β pathway often functions as a tumor promoter, and it has been associated with increased metastasis [38]. Our studies with HNSCC cell lines support a role for the TGF-β pathway as being pro-tumorigenic, in that it serves as an auxiliary growth factor pathway. Moreover, our studies show that TGF-β2 is upregulated at the transcriptional level in response to EGFR and FGFR-specific TKIs (Fig 2) and that inhibition of this pathway leads to decreased cell growth (Figs 3–5). TGF-β is secreted as a latent protein that requires extracellular activation prior to binding to the TGFβRs and inducing downstream signaling [35,39]. It has been reported that thrombospondin-1 (THBS1), a TKI-inducible gene (Fig 1), can participate in activation of latent TGF-β2 [28,40]. SPARC, another TKI-induced gene identified in all three cell lines tested has been shown to interact with THBS1 and also participate in extracellular TGF-β activation [29,41]. Thus, the HNSCC cell line response to TKI treatment involves the ligand TGF-β2, and distinct genes involved in the activation of the latent protein. Taken together, our data suggest a model whereby TGF-β2 functions as an inducible signal pathway that controls the intrinsic sensitivity to EFGR and FGFR inhibitors.

Furthermore, signaling downstream of the TGFβRs is also complex, where Smad signaling is considered the canonical signaling pathway [42,43]. In an effort to show changes in signaling downstream of TGF-β2, we measured phospho-Smad2 in UMSCC25 cells treated with control, 0.1μM gefitinib, 0.3μM AZD4547, or the combination for 4 days. However, an immunoblot revealed no change in phosho-Smad2 between the different treatment groups (S3 Fig). Thus, one or more non-canonical signaling pathways may be activated downstream of the TGFβRs when TGF-β2 is induced. Non-canonical TGFβR signaling pathways include MAPK pathways, the PI3K/Akt pathway, and Rho-like GTPase signaling pathways [44,45]. Attempts to dissect non-canonical signaling downstream of TGFβRs becomes difficult in our studies because the use of TKIs in this study also affect many of these pathways. Thus, elucidation of signaling pathways induced by TGF-β2 in HNSCC cells will require further investigation.

We recently demonstrated the existence of a signaling network involving EGFR, ERBB2, FGFRs, and MET in HNSCC cell lines where synergistic growth inhibition can be achieved with combinations of TKIs inhibiting all these RTKs [24]. Our present study indicates that the TGF-β2-TGFβR pathway adds to the mix, or network, of pathways driving growth in these HNSCC cell lines and may also function as a TKI-inducible resistance mechanism, although this will require extensive *in vivo* testing with xenografts in mice. Relative to lung cancers, HNSCC is noted for a general lack of somatic mutations and gene rearrangements in RTKs [2,46]. Rather, we hypothesize that complex RTK networks may serve as the growth driver in this cancer. Indeed, the existence of RTK co-activating networks is observed in multiple cancer types [47]. Simultaneous inhibition of TGF-β2-TGFβR and RTK signaling in HNSCC yields greater anti-tumor effects relative to blockade of either alone (Figs 3–5). Therefore, our data support the input from the TGF-β2-TGFβR pathway into this network.

While cetuximab is approved for treatment of HNSCC in conjunction with radiation and/or cytotoxic therapy [13], monotherapy with this inhibitor has not yielded cures. The existence of signaling networks in HNSCC rather than dominant mutated oncogenic drivers is likely to render monotherapy strategies ineffective. Rather, combination therapy with cocktails of TKIs and other targeted therapeutics is being put forward as a strategy for long-term control of cancers [48,49]. In this regard, TGFβR inhibitors might be considered as agents to include in combinations of targeted therapeutics for treatment of HNSCC.

## Supporting Information

S1 Fig HNSCC cells express TGFβRI and TGFβRII. qRT-PCR was performed on HNSCC samples to determine basal expression of A. TGFβRI and B. TGFβRII mRNA levels. Expression was normalized to GAPDH.
(PDF)

S2 Fig RNAi-mediated silencing of TGF-2 inhibits clonogenic growth in UMSCC25 cells. The data from Fig 3 is reorganized and graphed to display only gefitinib and the shRNA values. A. shTGFB2.4 and B. shTGFB2.5; ****denotes a p-value<0.0001; ***denotes a p-value of 0.002.
(PDF)

S3 Fig Phospho-Smad2 is not increased in TKI-treated UMSCC25 cells. UMSCC25 cells were treated for 4 days with DMSO-control, 0.3μM AZD4547, 0.1μM gefitinib, or the combination. Cell lysates were submitted to SDS-PAGE, and filters were probed for pSmad2 and total Smad2/3. No increase in pSmad2 was observed after incubation with TKIs. Na^+^/K^+^ ATPase was used as a loading control.
(PDF)

S4 Fig Original Immunoblots. These are the original, uncropped western blots with the ladders that are seen in S3 Fig.
(PDF)
