# Supplementary figures and images for: An Inducible TGF-β2-TGFβR Pathway Modulates the Sensitivity of HNSCC Cells to Tyrosine Kinase Inhibitors Targeting Dominant Receptor Tyrosine Kinases
Source: PLoS One. 2015 May 6;10(5):e0123600. doi: 10.1371/journal.pone.0123600 (PMC4422719; doi:10.1371/journal.pone.0123600)

**A**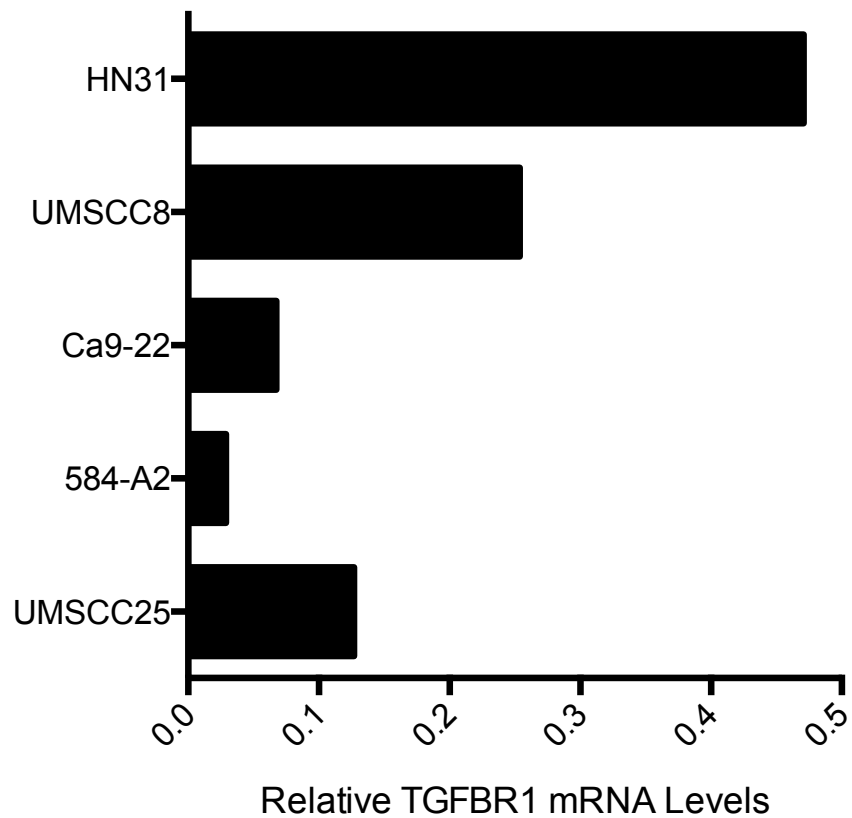**B**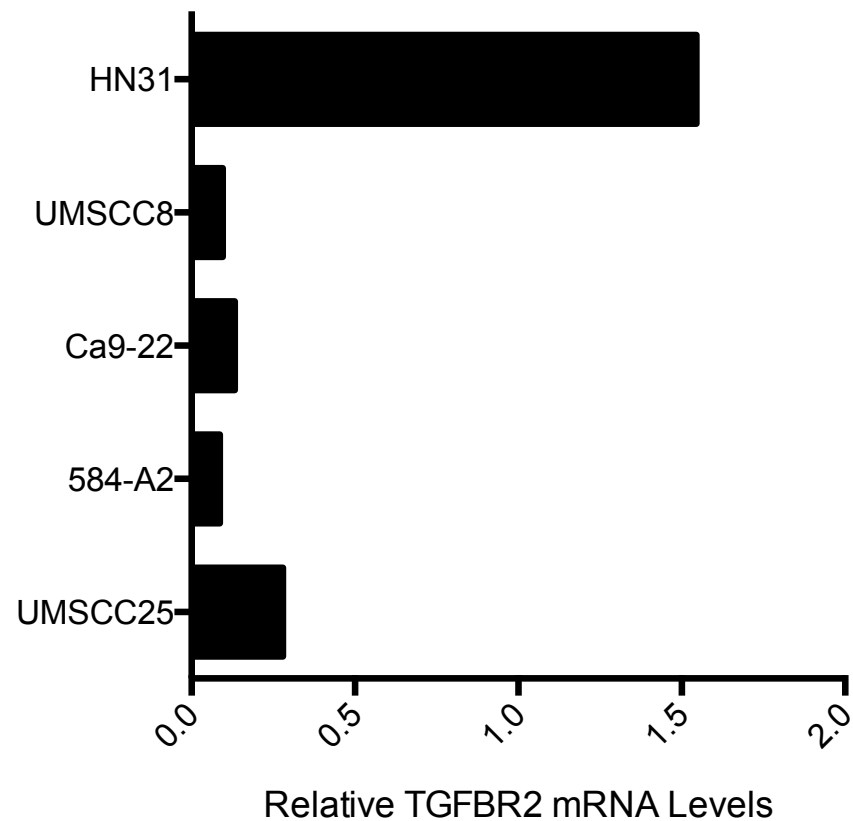

Supplement: S1 Fig — qRT-PCR was performed on HNSCC samples to determine basal expression of A. TGFβRI and B. TGFβRII mRNA levels. Expression was normalized to GAPDH. (PDF) [file pone.0123600.s001.pdf]

**A**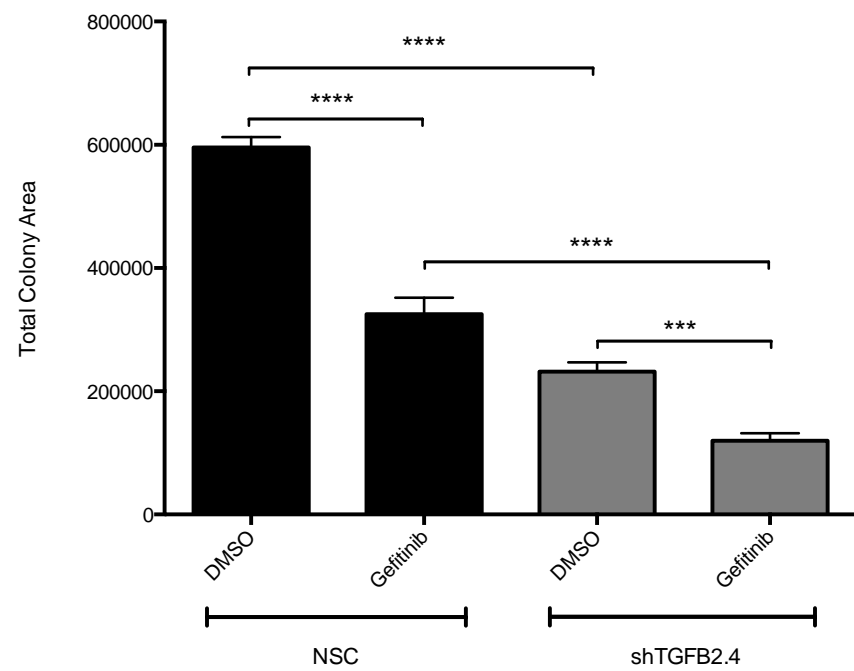**B**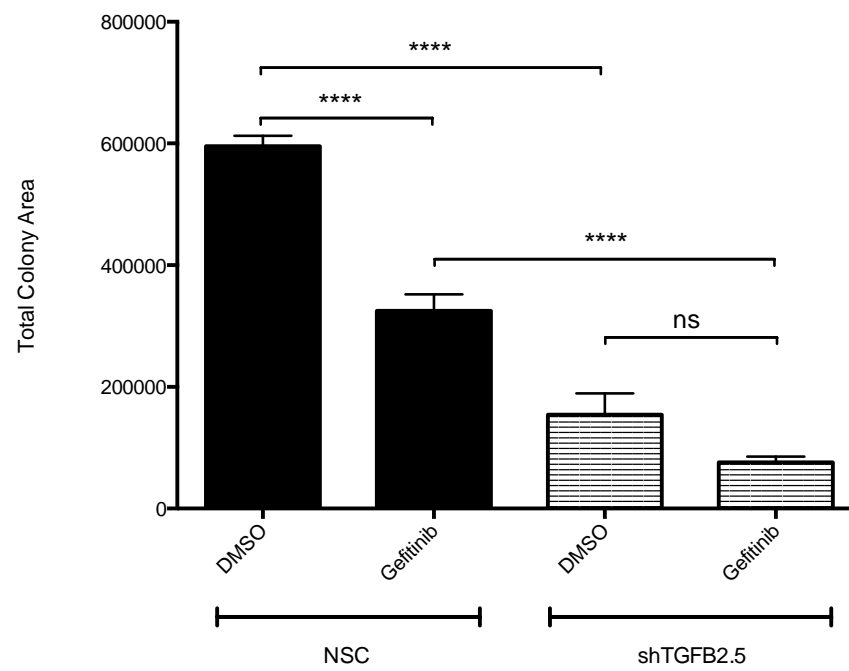

Supplement: S2 Fig — The data from Fig 3 is reorganized and graphed to display only gefitinib and the shRNA values. A. shTGFB2.4 and B. shTGFB2.5; ****denotes a p-value<0.0001; ***denotes a p-value of 0.002. (PDF) [file pone.0123600.s002.pdf]

**UMSCC25**

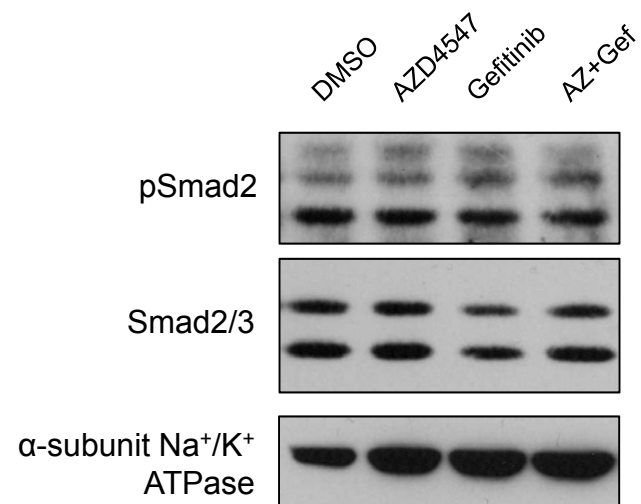

Supplement: S3 Fig — UMSCC25 cells were treated for 4 days with DMSO-control, 0.3μM AZD4547, 0.1μM gefitinib, or the combination. Cell lysates were submitted to SDS-PAGE, and filters were probed for pSmad2 and total Smad2/3. No increase in pSmad2 was observed after incubation with TKIs. Na+/K+ ATPase was used as a loading control. (PDF) [file pone.0123600.s003.pdf]
