# Supplementary material for: An Inducible TGF-β2-TGFβR Pathway Modulates the Sensitivity of HNSCC Cells to Tyrosine Kinase Inhibitors Targeting Dominant Receptor Tyrosine Kinases
Source: PLoS One. 2015 May 6;10(5):e0123600. doi: 10.1371/journal.pone.0123600 (PMC4422719; doi:10.1371/journal.pone.0123600)
Supplement: S4 Fig — These are the original, uncropped western blots with the ladders that are seen in S3 Fig. (PDF) [file pone.0123600.s004.pdf]

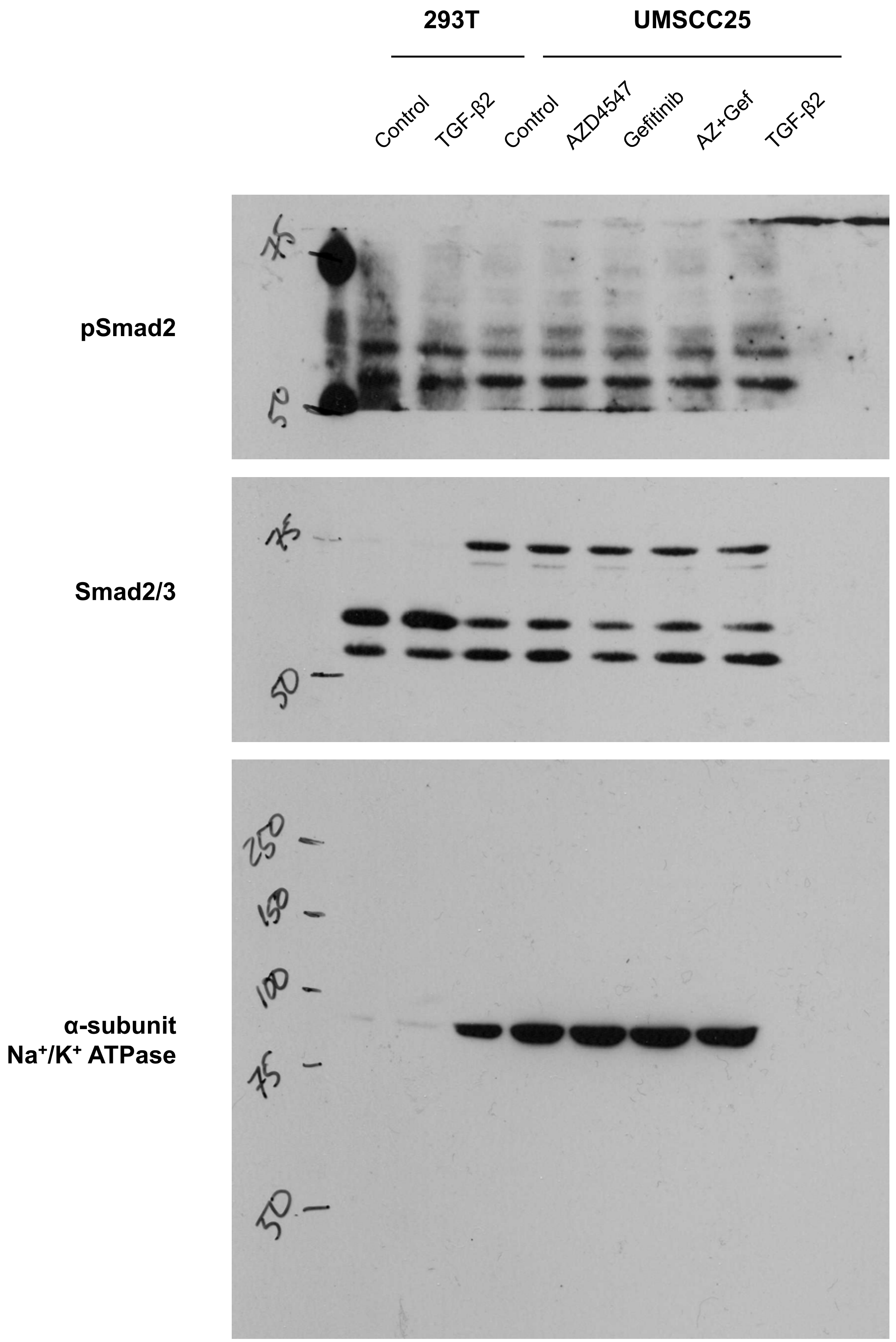
293T
UMSCC25
Control
TGF-β2
Control
AZD4547
Gefitinib
AZ+Gef
TGF-β2
pSmad2
Smad2/3
α-subunit
Na+/K+ ATPase
75
50
250
150
100
